# Neurodevelopmental Outcome and Adaptive Behavior in Preterm Multiples and Singletons at 1 and 2 Years of Corrected Age

**DOI:** 10.3389/fpsyg.2020.01653

**Published:** 2020-07-08

**Authors:** Chiara Squarza, Laura Gardon, Maria Lorella Giannì, Andrea Frigerio, Silvana Gangi, Matteo Porro, Fabio Mosca, Odoardo Picciolini

**Affiliations:** ^1^Fondazione IRCCS Ca’ Granda Ospedale Maggiore Policlinico, Neonatal Intensive Care Unit (NICU), Milan, Italy; ^2^Department of Clinical Science and Community Health, University of Milan, Milan, Italy; ^3^Pediatric Physical Medicine and Rehabilitation Unit, Fondazione IRCCS Ca’ Granda Ospedale Maggiore Policlinico, Milan, Italy

**Keywords:** neurodevelopmental outcome, behavioral outcome, multiple birth, extremely preterm, Griffiths mental development scales

## Abstract

**Background:**

Recent literature has investigated the role of multiple birth on neurodevelopmental outcomes of premature infants, especially extremely preterm ones. Multiple gestations are often associated to increased neurodevelopmental disability. Actually, research findings are controversial.

**Objective:**

To compare the neurodevelopmental and behavioral outcomes of multiples and singletons in a cohort of preterm infants ≤28 weeks gestational age at 1 and 2 years of corrected age.

**Methods:**

The study included 86 infants, born from January 2014 to September 2017 and enrolled in the follow-up program provided at authors’ Institution. Exclusion criteria included: major brain lesions and malformations, severe neuro-sensorial deficits, genetic syndromes, single-twin survivors. Thirty four multiples were compared to 52 singletons, using the Griffiths Mental Development Scales and the Child Behavior Checklist 1½–5. Statistical analysis was based on ANOVA techniques to test group differences. A *p* < 0.05 was considered statistically significant.

**Results:**

The neurodevelopmental outcomes of multiples and singletons at 1 and 2 years of corrected age did not significantly differ at a general level (*p* > 0.05). Multiples showed significantly lower mean scores than singletons at 1 year in Locomotor (87.15 ± 11.94 vs. 92.48 ± 11.59) and Personal-Social (84.88 ± 10.25 vs. 89.63 ± 8.19) subscales. Considering the behavioral outcomes, higher rates of externalizing problems were observed in multiples at 2 years (54.27 ± 9.64 vs. 49.31 ± 10.39).

**Conclusion:**

The slightly lower neurodevelopmental outcome showed by multiples, especially in the gross-motor and personal-social domains at 1 year, might be related to the specific environmental condition they experience. Multiple birth may affect mother’s sensitivity to infant’s needs and infant’s acquisition of emotional and behavioral regulation. This affects the separation process and the acquisition of the independent walking and other gross-motor skills. Being multiples might also induce an hyperstimulation and this could explain their higher vulnerability to externalizing problems (impulsiveness, hyperactivity, attention deficits). Additionally, males are more affected by the multiple condition than females.

## Introduction

In the last decades multiple births have exponentially increased, largely due to the diffusion of assisted reproductive technologies (Asztalos et al., 2001; Garg et al., 2010; Klebanoff and Keim, 2011). Multiple births carry several associated risks, such as monochorionicity, fetal death of the co-twin, abnormal placental communication, birth weight discordance and intrauterine growth restriction (Victoria et al., 2001; Shinwell et al., 2009). Moreover, many multiple pregnancies also result in premature births, increasing the number of infants born at extremely low gestational age or extremely low birth weight (ELBW) (Heino et al., 2016), who are widely acknowledged for being at higher risk of perinatal mortality and neurodevelopmental sequelae.

Furthermore, multiple birth poses important challenges both on parenting and on infants’ emotional and social development.

Mothers of twins struggle to achieve emotional attunement and sensitive mothering with each infant while they tend to develop an early preference towards one infant immediately after birth (Minde et al., 1990).

Besides biological and educative determinants, Zazzo (1976) identifies another important factor affecting twins’ development, the “twin situation.” The author posits that there is a system of relationships between the twins which influences their emotional and relational development. This “couple-effect” has both negative and positive effects, isolating twins from the environment and hindering their reciprocal separation process but also generating complementary roles which gradually help the partners’ differentiating from one another.

Although these are important findings, the association of multiple birth with the neurodevelopmental outcome of infants born preterm is a controversial issue, with previous published studies reporting inconsistent results. Several authors have demonstrated increased perinatal mortality and morbidity for multiple infants compared to singletons of same gestational age at birth (Papiernik et al., 2010; Petit et al., 2011), including those born before 26 weeks of gestational age (Ingram Cooke, 2010): multiples would be at greater risk for cerebral palsy (CP), cognitive deficit, learning disabilities and language impairment (Wadhawan et al., 2011; Porta et al., 2019).

According to other studies, the rates of cerebral palsy, major sequelae, and cognitive impairment would be similar in triplets and twins compared with singleton infants, even at lower gestational ages (Yee et al., 2008; Ray and Ward Platt, 2009; Yeo et al., 2015) or after adjusting for birth weight (Hajnal et al., 2005).

Furthermore literature has assessed neurodevelopmental outcome of multiples in comparison to singletons, primarily focusing on major disabilities and neuromotor deficits such as cerebral palsy, whereas the development of minor neurodevelopmental deficits and behavioral difficulties according to being either multiples or singletons has been poorly explored.

The aim of this study was to examine the neurodevelopmental and behavioral outcomes at 1 and 2 years of corrected age of a cohort of preterm singletons and multiples born at ≤28 weeks gestational age.

## Materials and Methods

### Participants

We implemented a single-center longitudinal cohort study. The study was approved by the Ethics Committee of the Fondazione IRCCS Ca’ Granda Ospedale Maggiore Policlinico and written informed consent was gathered from all the infants’ parents.

All the preterm infants consecutively born from January 2014 to September 2017 and enrolled after discharge in the follow-up program provided at authors’ Institution were eligible for the study.

Inclusion criterion was having a gestational age ≤28 weeks. Exclusion criteria included: major brain injuries detected on cerebral ultrasound (Intraventricular hemorrhage > grade 2 according to Papile et al. (1978), periventricular leukomalacia grade 2–4); severe sensory deficits (blindness, deafness); major congenital malformations and/or genetic syndromes. Single-twin survivors were also excluded since we aimed to investigate the environmental and relational effects of having a co-twin on preterm’s neurodevelopmental outcome.

### Procedure

Infants were recruited at 1 year of corrected age in occasion of the scheduled follow-up visit.

According to standard follow-up procedure, all the infants underwent the same follow-up evaluations.

Neurodevelopmental outcome at 1 and 2 years of corrected age was assessed using the Griffiths Mental Development Scales Revised, administered by two trained and qualified examiners. The Child Behavior Checklist 1 ½–5 was also administered to the infants’ mothers during the 2 years of corrected age follow-up visit.

The baseline characteristics of the sample were collected from the infants’ computerized medical charts.

Recorded data included: gestational age (GA), birth weight, small for gestational age (SGA, birth weight <10th centile for gestational age according to the Fenton Growth Chart (Fenton, 2003), gender, mode of delivery, length of hospital stay, duration of invasive mechanical ventilation or nasal continuous positive airway pressure (NCPAP).

The occurrence of Intraventricular Haemorrhage (IVH), Bronchopulmonary Dysplasia (BPD) and Retinopathy of Prematurity (ROP) were also collected.

Corrected age was calculated up to 24 months of life, from the chronological age adjusting for gestational age. Mothers’ nationality and education were recorded. Mothers’ educational level was considered an indicator of socioeconomic status and classified by a 3-point scale, with 1 indicating primary or intermediate school (≤8 years), 2 secondary school (9–13 years), and 3 university degree (>13 years).

### Assessment Tools

#### Neurodevelopmental Assessment

Neurodevelopmental outcome at 1 and 2 years of corrected age was examined by the validated Italian translation of the Griffiths Mental Development Scales Revised (Battaglia and Savoini, 2007), administered by two qualified examiners. The Scale consist of 5 subscales, which focus on the Locomotor, Personal-Social, Hearing and Language, Eye and Hand Coordination and Performance domains. Standardized General Quotient (mean 100, SD 12) and Sub-quotients (mean 100, SD 16) can be calculated. Accordingly, a typical development is expressed by a General Quotient of 88 or more and a Sub-quotient of 84 or more. By contrast, developmental delay is yield by a General Quotient of 87 or lower and a Sub-quotient of 83 or lower. As Italian normative data of the Griffiths Mental Development Scales Revised are not available, the 1996 UK norms were used (Griffiths and Huntley, 1996). Statistical evaluation of the test found the reliability of the tool to be adequate. The internal consistency of the items resulted in a correlation coefficient of 0.95 for the General Quotient and of 0.85–0.88 for single subscales.

#### Behavioral Assessment

Behavioral outcome was assessed using the Child Behavior Checklist 1½–5 (Achenbach and Rescorla, 2000), which measures behavioral and emotional symptoms for children ranging from 18 months to 5 years by means of parental reports. The CBCL yields a total scale of behavioral and emotional problems and separated internalizing and externalizing domains. According to the T-scores the behavior is categorized as normal (*T* < 65), borderline (*T* = 65–69), and clinical (*T* ≥ 70). The CBCL internalizing, externalizing, and total problems scales have demonstrated strong psychometric properties. Internal consistency of the items ranges from 0.72 to 0.94 depending on scale.

### Statistical Analysis

Descriptive statistics were calculated for perinatal and socio-demographic data, neonatal morbidity and neurodevelopmental outcome.

Differences between multiples and singletons were calculated by means of Fisher’s exact test for categorical variables and Mann-Whitney *U*-test for continuous variables.

ANOVA techniques were used to test group differences in GMDS-R and CBCL 1 ½–5 mean scores.

The IBM SPSS 21.0 (IBM Corp., Armonk, NY, United States) software package was used and *p* < 0.05 set as significance level.

## Results

A total of 186 infants (64.5% singletons and 35.5% multiples) with gestational age ≤28 weeks were admitted to NICU Fondazione IRCCS Ca’ Granda Ospedale Maggiore Policlinico during the study period.

**FIGURE 1:**
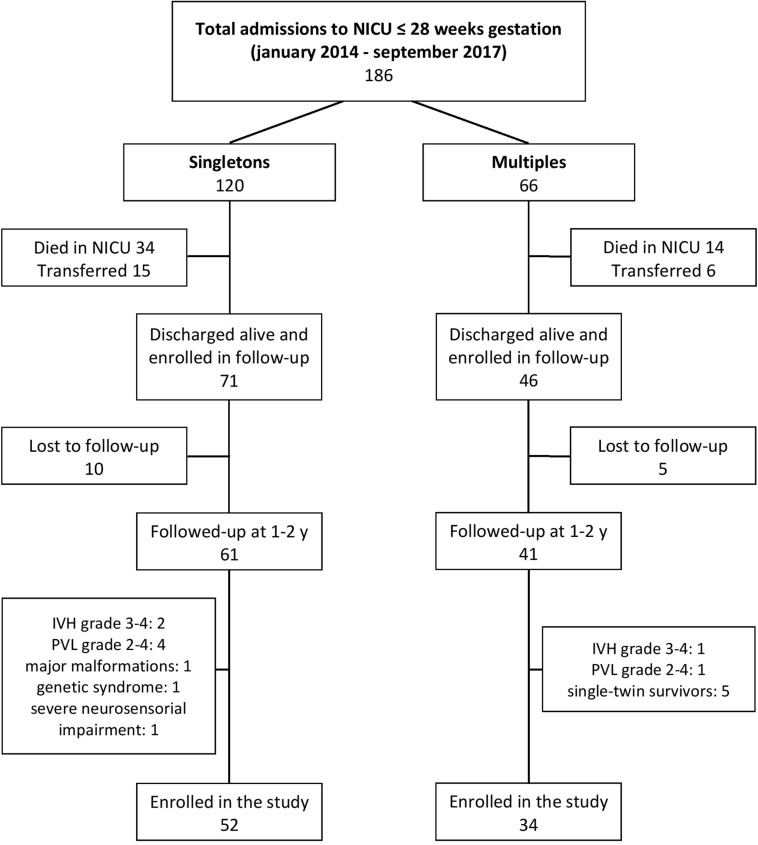
Flow chart of the study.

Among them, 117 (60.7% singletons and 39.3% multiples) were discharged alive and enrolled in the follow-up program. Of these, 102 (59.8% singletons and 40.2% multiples) returned for the 1–2 years follow-up visits and 86 (60.5% singletons and 39.5% multiples) met the inclusion criteria of the study and were assessed at 1 and 2 years of corrected age. Figure 1 shows the flow chart of the study.

In our study sample 10 (11.6%) infants were outborn, equally distributed between singletons (11.5%) and multiples (11.8%). Of the multiple births, 24% were triplets.

Mothers’ and infants’ characteristics were comparable between the two groups (Table 1), except for the rates of assisted conception (9.6 vs. 47.1% in singletons and multiples, respectively, *p* < 0.000) and IVH grades 1–2 (1.9 vs. 8.8% in singletons and multiples, respectively, *p* < 0.05).

**TABLE 1:** Baseline characteristics of singletons and multiples groups.

	**Singletons (*n* = 52)**	**Multiples (*n* = 34)**	***p*-value**
**Infants characteristics**			
Gestational age at birth (weeks), mean ± SD	26.6 ± 1.4	26.7 ± 1.2	0.956^a^
Birth weight (g), mean ± SD	917.1 ± 263.7	905.2 ± 197.9	0.989^a^
Male, *n* (%)	26 (50.0%)	16 (47.1%)	0.828^b^
Small for gestational age, *n* (%)	9 (17.3%)	2 (5.9%)	0.188^b^
Assisted conception, *n* (%)	5 (9.6%)	16 (47.1%)	0.000^b^*
Cesarean section, *n* (%)	38 (73.1%)	27 (79.4%)	0.611^b^
Mechanical ventilation (days), mean ± SD	8.27 ± 12.2	12.6 ± 17.2	0.240^a^
Total length of hospital stay (days), mean ± SD	99.5 ± 49.5	114.2 ± 59.8	0.202^a^
Sepsis, *n* (%)	17 (32.7%)	15 (44.1%)	0.362^b^
ROP (stage III-IV), *n* (%)	7 (13.5%)	5 (14.7%)	0.439^b^
Bronchopulmonary dysplasia, *n* (%)	21 (40.4%)	16 (47.1%)	0.65^b^
IVH 1-2, *n* (%)	1 (1.9%)	3 (8.8%)	0.046^b^*
Older siblings, *n* (%)	16 (30.8%)	6 (17.6%)	0.212^b^
**Mothers characteristics**			
Mothers age at childbirth (years), mean ± SD	32.5 ± 5.6	33.7 ± 4.6	0.460^a^
Mothers nationality (not Italian), *n* (%)	25 (48.1%)	9 (26.5%)	0.282^b^
Mothers schooling (university degree), *n* (%)	22 (42.3%)	18 (52.9%)	0.242^b^

^*a*^Mann-Whitney U-test. ^*b*^Fisher’s exact test. Bolded values are statistically significant (*p* < 0.05). *means that those values are statistically significant (*p* < 0.05).

The neurodevelopmental outcomes of the two groups are described in Tables 2–5.

**TABLE 2:** Neurodevelopmental outcome of singletons and multiples at 1 year.

***Griffiths Mental Development Scales***	**Singletons (*n* = 52)**	**Multiples (*n* = 34)**	***p*-value**
	**Mean ± SD**	**Mean ± SD**	
General quotient	90.00 ± 8.97	86.68 ± 9.56	0.105
Locomotor	92.48 ± 11.59	87.15 ± 11.94	0.042*
Personal-social	89.63 ± 8.19	84.88 ± 10.25	0.020*
Hearing and language	90.96 ± 6.85	89.03 ± 6.49	0.195
Eye and hand coordination	89.85 ± 10.19	88.26 ± 10.11	0.482
Performance	91.50 ± 9.69	88.68 ± 9.70	0.191

Bolded values are statistically significant (*p* < 0.05). *means that those values are statistically significant (*p* < 0.05).

**TABLE 3:** Neurodevelopmental outcome of male vs. female singletons and multiples at 1 year.

**Males**			

***Griffiths Mental Development Scales***	**Singletons (*n* = 26)**	**Multiples (*n* = 16)**	***p*-value**
	**Mean ± SD**	**Mean ± SD**	
General quotient	90.12 ± 8.17	82.25 ± 11.36	0.013*
Locomotor	93.35 ± 9.25	81.13 ± 11.59	0.001*
Personal-social	88.92 ± 8.52	80.81 ± 11.86	0.014*
Hearing and language	91.62 ± 6.47	86.81 ± 8.29	0.042*
Eye and hand coordination	88.35 ± 10.32	83.19 ± 11.08	0.134
Performance	90.96 ± 10.51	83.81 ± 11.40	0.045*

**Females**			

***Griffiths Mental Development Scales***	**Singletons (*n* = 26)**	**Multiples (*n* = 18)**	***p*-value**
	**Mean ± SD**	**Mean ± SD**	

General quotient	89.88 ± 9.87	90.61 ± 5.36	0.778
Locomotor	91.62 ± 13.67	92.50 ± 9.68	0.814
Personal-social	90.35 ± 7.97	88.50 ± 7.12	0.434
Hearing and language	90.31 ± 7.28	91.00 ± 3.52	0.711
Eye and hand coordination	91.35 ± 10.04	92.78 ± 6.64	0.600
Performance	92.04 ± 8.99	93.00 ± 5.09	0.684

Bolded values are statistically significant (*p* < 0.05). *means that those values are statistically significant (*p* < 0.05).

**TABLE 4:** Neurodevelopmental outcome of singletons and multiples at 2 years.

***Griffiths Mental Development Scales***	**Singletons (*n* = 52)**	**Multiples (*n* = 34)**	***p*-value**
	**Mean ± SD**	**Mean ± SD**	
General quotient	87.40 ± 12.76	85.12 ± 14.30	0.441
Locomotor	94.27 ± 9.69	92.71 ± 11.22	0.494
Personal-social	87.02 ± 10.77	84.12 ± 14.04	0.283
Hearing and language	85.62 ± 14.38	82.62 ± 17.63	0.390
Eye and hand coordination	91.29 ± 11.94	88.09 ± 12.91	0.243
Performance	90.58 ± 11.09	87.88 ± 14.01	0.324

**TABLE 5:** Neurodevelopmental outcome of male vs. female singletons and multiples at 2 years.

**Males**			

***Griffiths Mental Development Scales***	**Singletons (*n* = 26)**	**Multiples (*n* = 16)**	***p*-value**
	**Mean ± SD**	**Mean ± SD**	
General quotient	85.00 ± 14.94	78.31 ± 16.25	0.181
Locomotor	95.58 ± 7.50	87.38 ± 13.11	0.013*
Personal-social	84.35 ± 13.02	76.31 ± 14.89	0.073
Hearing and language	81.92 ± 16.63	76.06 ± 18.72	0.297
Eye and hand coordination	89.23 ± 13.64	83.00 ± 15.93	0.185
Performance	88.65 ± 12.73	80.44 ± 16.28	0.075

**Females**			

***Griffiths Mental Development Scales***	**Singletons (*n* = 26)**	**Multiples (*n* = 18)**	***p*-value**
	**Mean ± SD**	**Mean ± SD**	

General quotient	89.81 ± 9.87	91.17 ± 9.06	0.645
Locomotor	92.96 ± 11.49	97.44 ± 6.50	0.143
Personal-social	89.69 ± 7.24	91.06 ± 8.89	0.579
Hearing and language	89.31 ± 10.83	88.44 ± 14.76	0.824
Eye and hand coordination	93.35 ± 9.81	92.61 ± 7.33	0.789
Performance	92.50 ± 9.02	94.50 ± 6.99	0.434

Bolded values are statistically significant (*p* < 0.05). *means that those values are statistically significant (*p* < 0.05).

At 1 year, a significant difference emerged between the two groups in Locomotor and Personal-Social subscales, while at 2 years no statistically significant differences were found.

At 2 years, the Locomotor subscale had the highest mean scores, while the Hearing and Language and Personal-Social subscales had the lowest ones.

At both ages, the General Quotient and Subscales mean scores were in the average or low-average range for both groups, with multiples showing slightly lower mean scores than singletons in all subscales.

Further discrepancies in neurodevelopmental outcomes emerged considering males and females separately. At 1 year, male multiples showed significantly lower scores than male singletons both in general quotient and in almost all subscales (Table 3). At 2 years differences between male multiples and singletons reduced, with male multiples showing significantly worse outcomes than singletons only in Locomotor subscale (Table 5).

By contrast, no differences were found between female singletons and female multiples either at 1 and 2 years.

Considering the behavioral outcomes overall, Internalizing, Externalizing and Total Scale mean scores were within the normal range in both groups (Table 6). Multiples had significantly higher externalizing scores than singletons. No differences were found in any scales between males and females (Table 7).

**TABLE 6:** Behavioral problems of singletons and multiples groups at 2 years of corrected age.

***Child Behavior Checklist 1½–5 (T-scores)***	**Singletons (*n* = 45)**	**Multiples (*n* = 30)**	***p*-value**
	**Mean ± SD**	**Mean ± SD**	
Total	50.89 ± 9.29	53.10 ± 10.47	0.341
Internalizing problems	51.69 ± 9.69	49.40 ± 10.12	0.328
Externalizing problems	49.31 ± 10.39	54.27 ± 9.64	0.041*

Bolded values are statistically significant (*p* < 0.05). *means that those values are statistically significant (*p* < 0.05).

**TABLE 7:** Behavioral problems of male and female singletons and multiples groups at 2 years of corrected age.

**Males**			

***Child Behavior Checklist 1½–5 (T-scores)***	**Singletons (*n* = 20)**	**Multiples (*n* = 13)**	***p*-value**
	**Mean ± SD**	**Mean ± SD**	
Total	51.40 ± 9.59	56.92 ± 5.44	0.069
Internalizing problems	51.60 ± 8.39	51.92 ± 6.26	0.906
Externalizing problems	52.00 ± 11.68	57.69 ± 3.68	0.100

**Females**			

***Child Behavior Checklist 1½–5 (T-scores)***	**Singletons (*n* = 25)**	**Multiples (*n* = 17)**	***p*-value**
	**Mean ± SD**	**Mean ± SD**	
Total	50.48 ± 9.23	50.18 ± 12.47	0.928
Internalizing problems	51.76 ± 10.78	47.47 ± 12.13	0.236
Externalizing problems	47.16 ± 8.91	51.65 ± 11.89	0.170

## Discussion

Our findings suggest that the neurodevelopmental outcomes of singletons and multiples at 1 and 2 years of corrected age are not significantly different at a general level. Multiples show slightly lower mean scores, which may have clinical significance, although the small study sample might limit the statistical power of comparisons. At a more analytic level, multiples show significantly lower mean scores than singletons in Locomotor and Personal-Social subscales at 1 year. Considering the behavioral outcomes, higher rates of externalizing problems are observed in multiples at 2 years.

We hypothesize that the lower scores in Locomotor and Personal-Social subscales at 1 year in multiples might be interconnected and both related to a specific environmental condition.

Being multiples might hinder the acquisition of emotional and behavioral regulation. A multiple birth may compromise the mother’s ability to reach an adequate affect attunement and to provide sensitive mothering to each infant (Szajnberg et al., 1989). Moreover, multiples have to share most part of the caregiving, experiencing fewer moments of one-to-one interaction with parents, which is essential to reach better regulation strategies (Feldman et al., 2004). Parents of multiples tend to enhance occasions of spontaneous interaction between twins, which are still immature and low regulated, and so not able to support each other’s behavioral adaptation (Theroux, 1989). This might lead to a higher vulnerability to externalizing problems at 2 years.

Triplet condition may represent a cumulative risk factor, in addition to prematurity and twin condition, for the achievement of self-regulation and behavioral adaptation. Compared to twins, a triplet birth is remarkably more stressful for parents, hindering maternal sensitivity and reducing the occasions of exclusive parenting for each child (Bendersky and Lewis, 1994). Literature reports that the constant competition for parental affection, attention and stimulation which triplet infants have to face negatively affects their emotional and cognitive development (Goshen-Gottstein, 1980; Bryan, 1992).

In our sample, the particular fragility in personal and social abilities seems to affect also gross motor skills. In particular, poor emotional regulation may hinder the separation process, delaying the acquisition of independent walking. The low scores in the Personal-Social subscale reveal poor personal self-sufficiency and a difficulty in separating from parents. Often the child seems capable of walking by himself but is afraid and remains emotionally dependent on the adult.

Consistently with literature (Grether et al., 1993; Bonellie et al., 2005), in our findings gender had a significant effect on neurodevelopmental outcomes, with male multiples having worse outcomes than female multiples.

We hypothesize that males, generally considered at higher risk of neurodevelopmental deficits, are even more affected by the multiple condition. By contrast, female gender might have a buffer effect, balancing the consequences of multiple birth on neurodevelopmental outcomes. However, gender effects tend to decrease over time, reducing considerably at 2 years, considering both neurodevelopmental domains and adaptive behaviors.

The lack of significant differences between multiples’ and singletons’ neurodevelopmental outcomes at a global level in our cohort is consistent with other studies.

Gnanendran et al. (2015) reported that multiples (<29 weeks) and similarly preterm singletons at 2–3 years of corrected age did not have significantly higher rates of neurodevelopmental impairment. Similarly, Eras et al. (2013) did not find any significant association between multiple birth in preterm infants (≤32 weeks) and increased risk of neurodevelopmental deficits at 12–18 months’ corrected age.

However, population-based studies evaluating singletons and multiples have shown varying outcomes.

Studying a cohort of ELBW infants at 18–22 months of corrected age, Wadhawan et al. (2009) highlighted that twins, triplets and higher-order multiples had worse neurodevelopmental outcome compared to singletons.

Most published studies on preterm multiples and singletons mainly investigate neonatal morbidity and mortality and data on neurodevelopmental outcomes primarily focus on neuromotor deficits (Asztalos et al., 2001). Few published studies have provided a more comprehensive description of neurodevelopmental domains, including emotional ones, for very preterm multiples with low gestational age. A strength of our study is that it focused on infants’ neurodevelopmental and behavioral outcomes, providing an analytic profile of all psychomotor domains.

### Limitations and Future Directions

A limitation of our study might be the insufficient power for specific comparisons in any psychomotor domain, given the small study sample. Further studies are needed to compare cognitive and behavioral outcomes of singletons and multiples at school age or with other specific assessment tools.

Furtherly, the significative higher rate of assisted conception in multiples group raises questions on the effects of artificial reproduction techniques on preterm outcomes. Previous studies highlighted better survival rates in NICU for preterm infants following assisted conception (Garg et al., 2010). Further research should address the impact of assisted conception also on neurodevelopmental outcomes of preterm singletons and multiples.

## Conclusion

Our findings suggest that very preterm singletons and multiples with gestational age ≤28 weeks have similar neurodevelopmental outcome at 1 and 2 years corrected age. On average, multiples had slightly lower mean scores than singletons, but the difference was not significant. However, multiples showed a higher vulnerability to emotional and behavioral dysregulation at 1 year. We hypothesize that these early difficulties in self-regulation might lead to symptoms of impulsiveness, hyperactivity, and attention deficits at 2 years of age.

Our findings have key implications for preventive services and follow-up clinics. It is important to follow multiples beyond the infancy period to verify if the developmental differences in motor and personal-social abilities found in the first year attenuate or improve as infants grow, and it is essential to investigate what ages are at higher risk as to provide timely interventions.

## Data Availability Statement

The datasets presented in this article are not readily available because of privacy policies. Requests to access the datasets should be directed to odoardo.picciolini@policlinico.mi.it.

## Ethics Statement

The study design was approved by the Ethics Committee of the Fondazione IRCCS Ca’ Granda Ospedale Maggiore Policlinico and written informed consent was obtained from all the infants’ parents.

## Author Contributions

CS, LG, and AF conceptualized and designed the study, drafted the initial manuscript, and approved the final manuscript as submitted. MG, SG, and MP designed the data collection instruments, carried out the initial analyses, reviewed and revised the manuscript, and approved the final manuscript as submitted. FM and OP coordinated and supervised data collection, critically reviewed the manuscript, and approved the final manuscript as submitted. All authors approved the final manuscript as submitted and agree to be accountable for the content of the work.

## Conflict of Interest

The authors declare that the research was conducted in the absence of any commercial or financial relationships that could be construed as a potential conflict of interest.

## Abbreviations

AGA/SGA: Adequate/Small for Gestational Age
BPD: Bronchopulmonary dysplasia
ELBW: Extremely Low Birth Weight
EP: Extremely Preterm
GA: Gestational Age
IVH: Intraventricular hemorrhage
MRI: Magnetic Resonance Imaging
ROP: Retinopathy of Prematurity
SD: Standard Deviation.
